# IgG4 as a Biomarker in Graves' Orbitopathy

**DOI:** 10.1155/2021/5590471

**Published:** 2021-06-10

**Authors:** Michał Olejarz, Ewelina Szczepanek-Parulska, Daniela Dadej, Nadia Sawicka-Gutaj, Remigiusz Domin, Marek Ruchała

**Affiliations:** Department of Endocrinology, Metabolism and Internal Medicine, Poznan University of Medical Sciences, Przybyszewskiego St. 49, 60-355 Poznań, Poland

## Abstract

Immunoglobulin G4-related disease (IgG4-RD) is a chronic inflammatory disorder associated with fibrosis and abundant tissue lymphoplasmacytic infiltrations. It typically affects the pancreas, the salivary glands, and the retroperitoneal space. However, it might also involve multiple other organs, including the orbit and the thyroid. Recent studies have suggested that IgG4 plays a role in the pathophysiology of autoimmune thyroid diseases. This ultimately led to the establishment of new clinical entities called IgG4-related thyroid disease and thyroid disease with an elevation of IgG4. The aim of this paper is to describe the pathophysiological, histopathological, and clinical features of Graves' Disease (GD) and Graves' Orbitopathy (GO) with elevated IgG4 levels. Multiple studies have demonstrated higher IgG4 serum concentrations in GD patients than in healthy euthyroid controls. Depending on the studied population, elevated serum IgG4 levels occur in 6.4-23% (average: 10.3%) of all patients with GD, 8.3-37.5% (average: 17.6%) of patients with GO, and 0-9.8% (average: 5.4%) of patients with GD without GO, while GO patients comprise 37.5-100% (average: 65.8%) of all GD patients with elevated IgG4 levels. Characteristic features of GD with elevated IgG4 levels include lower echogenicity of the thyroid gland on ultrasound examination, peripheral blood eosinophilia, higher prevalence of orbitopathy, and better response to antithyroid drugs with a tendency to develop hypothyroidism when compared to patients with GD and normal levels of IgG4. Typical signs of GO accompanied by increased concentration of IgG4 include younger age at diagnosis, and more severe course of the disease with a higher Clinical Activity Score (CAS).. We strongly recommend considering the diagnosis of GO with elevated IgG4 in patients with an established diagnosis of GD, elevated serum IgG4 levels, and clinical features of ophthalmic disease overlapping with those of IgG4-related orbital disease.

## 1. Introduction

Human immunoglobulin G (IgG) subclasses were numbered in an order reflecting the time of their discovery, which also corresponds to their prevalence in plasma [1]. Thus, IgG4 is the last discovered and least abundant IgG subclass, but this does not make it the least important, especially since Hamano et al., for the first time, reported that sclerosing pancreatitis is related to high levels of immunoglobulin G4 (IgG4) [2]. In 2003, Kamisawa et al. proposed the term “IgG4-related autoimmune disease” as a systemic disease with multiorgan involvement [3]. The authors used various nomenclatures to describe conditions related to the presence of IgG4 until 2010 in Kanazawa, where it was unified as an “IgG4-related disease” with the consensus that it can affect various organs, including the thyroid and the eye with its adnexa and extraocular muscles [4].

Immunoglobulin G4-related disease (IgG4-RD) is a chronic inflammatory disorder associated with fibrosis and abundant lymphoplasmacytic infiltrations, often with obliterative phlebitis and storiform fibrosis within the affected tissues. IgG4-RD appears to underlie several fibroinflammatory disorders earlier regarded as idiopathic or isolated [5–7]. Typical IgG4-RD manifestations are pancreatitis, sialadenitis, retroperitoneal fibrosis, and cholangitis. However, IgG4-RD can involve the vast majority of organs, including the thyroid and the orbit [2, 8, 9].

The symptoms depend on the affected organ. On histopathology, this fibroinflammatory disease is characterized with specific findings such as lymphoplasmacytic infiltration of IgG4-positive plasma cells, obliterative phlebitis, and storiform fibrosis, in most cases accompanied by increased levels of serum IgG4 [10].

To date, an association between IgG4 and various other diseases like allergies, cancer, or rheumatoid arthritis has been revealed [11]. Until now, the relationship between IgG4, Graves' Disease, and Graves' Orbithopathy has been investigated in several studies; however, the data is limited.

The aim of this paper is to summarize current knowledge on the role of IgG4 as a biomarker of Graves' Disease and Graves' Orbitopathy.

## 2. IgG4 in the Pathogenesis of Inflammatory Eye Disease and Systemic Autoimmune Disease

Increased immunoglobulin G4 (IgG4) concentration is a common, though nonspecific, finding observed in most IgG4-RD patients [12]. High IgG4 concentration is also observed in allergic, rheumatic, and neoplastic diseases, being conditions characterized by prolonged immunization [1, 13]. Although the nomenclature implies a crucial role of IgG4 in this disorder, it is still unclear if IgG4 are pathogenic or appear secondary to the disease and reflect an anti-inflammatory reaction.

IgG4 is the least abundant class of IgG. Due to the specific structural features, IgG4 exerts a unique ability of Fab arm exchange [14]. Exchange of the Fab arms between the two antibodies results in the formation of monovalent bispecific molecules directed against two unrelated antigens. Consequently, IgG4 are unable to form immune complexes. IgG4 demonstrates a poor ability to activate the complement [15]. In allergic reactions, IgG4 prevents immunoglobulin E (IgE) from binding with mast cells, thus inhibiting their degranulation [16]. IgG4 also interferes with T helper type 2 (Th2) cells, reducing their proinflammatory actions. Shiokawa et al. investigated the pathogenic roles of IgG1 and IgG4 in mice [17]. Antibodies of both classes, obtained from IgG4-RD patients, were injected into mice leading to injuries in several organs with most detrimental effects caused by IgG1. Simultaneous injection of IgG4 reduced IgG1-related injuries. This would suggest rather an anti-inflammatory and tolerance-inducing action of IgG4. However, IgG4 in IgG4-RD were found to have an increased ability to bind and activate the complement, which may imply their pathogenic significance [18].

The direct cause of IgG4-RD remains unknown. Growing evidence suggests an autoimmune basis for the disease. Potential best-studied autoantigens include annexin A11, laminin 511, and galectin-3; however, their role requires clarification [19–21]. The mechanisms of both the innate and acquired immune responses are involved in the pathogenesis of IgG4-RD.

Exposure to foreign antigens activates circulating monocytes and basophils. In consequence, they produce the B cell-activating factor (BAFF) that induces B cell proliferation and differentiation towards IgG4-producing plasmablasts and plasma cells [22, 23]. In patients with IgG4-RD, the concentration of BAFF was found to be higher compared to healthy controls [24]. Activated monocytes differentiate into tissue macrophages. In IgG4-RD, the M2 macrophages, which participate in anti-inflammatory reactions and Th2-mediated response, dominate over M1 phenotype cells [25, 26]. Recently, interleukin-33 (IL-33), a cytokine that strongly activates innate immunity, was reported to promote macrophage polarization towards the M2 type and thus induce Th2 response underlying IgG4-RD [27]. The M2 macrophages were found to secrete IL-33 in patients with IgG4-RD [27, 28], as well as profibrotic factors such as interleukin-10 (IL-10), interleukin-13 (IL-13), and C-C Motif Chemokine Ligand 18 (CCL18) [26]. Hence, basophils and macrophages contribute to the increased IgG4 production, stimulation of Th2-mediated response, and fibrosis in the course of IgG4-RD. The role of other elements of innate immunity remains controversial [29].

T cells predominate in IgG4-RD tissue infiltrates and play an important role in its pathogenesis [30]. The latest research points towards CD4+ cytotoxic T lymphocytes (CD4+ CTL) as the key components of IgG4-RD immunopathogenesis [31, 32]. In IgG4-positive patients, CD4+ CTL express signaling lymphocytic activation molecule family member 7 (SLAMF7), a surface antigen allowing interactions with antigen-presenting B cells, and secrete granzyme A, interleukin-1*β*, interferon-gamma (IFN-*γ*), and transforming growth factor-*β*1 (TGF-*β*1) inducing local cytotoxicity, tissue damage, and fibrosis [31, 33]. High concentrations of clonally expanded CD4+ CTL in blood and affected organs were observed in active IgG4-RD. Both B cell depletion treatment with rituximab and glucocorticoid treatment improved the symptoms and decreased CD4+ CTL levels [31, 32]. This implies a central role of CD4+ CTL in IgG4-RD pathogenesis.

Other T cell subsets are also involved in IgG-RD pathogenesis. Both T helper type 2 cells and regulatory T cells (Treg) were reported to increase in IgG4-RD [34–36]. The activated Th2 cells secrete interleukin-4 (IL-4) and IL-13, which along with IL-10 and TGF-*β* produced by Treg are considered to drive tissue damage. The IL-13 and TGF-*β* promote myofibroblast differentiation and extracellular matrix protein synthesis [37], whereas IL-10 and IL-4 stimulate B lymphocyte differentiation towards IgG4-producing plasmablasts and plasma cells [38]. The follicular subsets of both the T helper and regulatory cells, mostly localized in the germinal centers (GC) [39], seem to substantially contribute to the IgG4-RD pathogenesis. Notably, ectopic GC were identified in IgG4-RD-affected tissues [40]. Increased T follicular regulatory cells and T follicular helper (Tfh) cells, especially Tfh2, were reported in IgG4-RD patients, compared to healthy controls, and correlated positively with serum IgG4 concentration and the number of involved organs [41–44]. Tfh cells decreased following glucocorticoid treatment, while the disease relapse resulted in their upturn [42, 45]. Tfh cells produce interleukin-21 (IL-21) that along with IL-4 promotes B cell differentiation towards plasmablasts and drives IgG4 class switching. IL-21 is essential for GC formation and sustainability [45–47]. Tfh cells obtained from IgG4-RD patients induced B cell proliferation and differentiation more potently than the Tfh cells from healthy individuals [42].

In IgG4-RD, the B cell subpopulations and their regulatory cytokines are altered. The aforementioned mechanisms result in the development of IgG4-producing plasma cells and their short-lived precursors—plasmablasts. Clonally expanded CD19^+^20^−^27^+^38^hi^ plasmablasts, producing IgG4, were found to increase in circulation and tissue infiltrates, compared to healthy individuals, and to correlate with the IgG4-RD activity and extension (number of affected organs) irrespective of IgG4 concentration. Plasmablasts declined after B cell depletion treatment with rituximab and glucocorticoid treatment, which resulted in clinical improvement, while in relapse, they reemerged [48–50]. B cells not only act by inducing autoantibodies and activating T cell response but also directly promote fibrosis by secreting cytokines such as fibroblast-stimulating platelet-derived growth factor-*β* (PDGF-*β*), enzymes modifying the extracellular matrix such as lysyl oxidases, or chemokines such as CCL4, CCL5, and CCL11 (C-C Motif Chemokine Ligands 4, 5, and 11) [51].

In several disorders, including myasthenia or pemphigus, the pathogenic role of IgG4 is well defined. Due to the complexity of IgG4-RD pathogenesis, the potential contribution of IgG4 in this disease remains unclear.

## 3. Role of IgG4 in Graves' Disease and Orbitopathy

### 3.1. Main Features of Graves' Orbitopathy and IgG4-Related Ophthalmic Disease

Graves' Disease (GD) is an autoimmune thyroid disease, where the most important clinical feature is hyperthyroidism [52]. It can also affect the eyes and orbital tissue in up to 40% of patients and cause an inflammatory eye disease called Graves' Orbitopathy (GO) [53]. From a clinical point of view, the typical signs of GO include eyelid retraction (most common sign—occurs in up to 98% of patients), lid lag, eyelid swelling and erythema, inflammation of the caruncle and plica, and orbital tissue and muscle swelling [54, 55]. Many conditions may mimic GO, including orbital pseudotumor; orbital neoplasms, both malignant (e.g., lymphomas) and benign; infections; orbital myositis; and inflammatory orbitopathy, e.g., granulomatosis with polyangiitis or sarcoidosis [56]. Orbitopathy can also occur in other autoimmune thyroid diseases like Hashimoto's thyroiditis, but it is exceptionally rare compared to the incidence rate of GD [57]. Therefore, the differential diagnosis in patients with eye proptosis or periorbital edema should be performed in each case. An important differential diagnosis for GO is IgG4-related ophthalmic disease (IgG4-ROD). IgG4-RD can possibly affect all kinds of tissues. Typical IgG4-ROD manifestation is dacryoadenitis—inflammation of lacrimal glands [58]. Other tissues and the ocular adnexa involved are extraocular muscles (especially the lateral rectus muscle), orbital fat and soft tissue (pseudotumor), eyelids, the trigeminal nerve (especially the infraorbital nerve), the optical nerve, and even orbital bones [59]. Even though elevated serum IgG4 levels and increased numbers of IgG4(+) plasma cells in tissues are considered to be a hallmark of IgG4-RD [60], recent studies suggest that IgG4 plays a role in the pathophysiology of autoimmune thyroid diseases, including GO. This ultimately led to the establishment of new clinical entities called IgG4-related thyroid disease (IgG4-RTD) and thyroid disease with an elevation of IgG4 (in those diseases, where elevated IgG4 levels are observed, but there is not enough histopathological or pathophysiologic evidence to classify them as IgG4-RTD). Riedel's thyroiditis is widely acknowledged as one of the typical IgG4-RD manifestations. However, the gross majority of other autoimmune thyroiditis diseases with an elevation of IgG4 or considered to be IgG-RTD seem to be organ-specific diseases with no systemic manifestations of IgG4-RD [61, 62]. Thus, during the differential diagnosis of orbitopathy, along with histopathological and biochemical analysis, it is also worth looking actively for systemic changes in other organs by performing imaging diagnostics in the form of CT, MRI, or PET-FDG examinations.

### 3.2. Pathophysiology of Graves' Orbitopathy

The pathophysiology of GO is very complex and still not fully understood. It involves complex interactions between a variety of immune cells, cytokines, interleukins, adhesion molecules, and growth factors and increased oxidative stress. A key role is being assigned to abnormally activated orbital fibroblasts, overexpression of the human leukocyte antigen-DR (HLA-DR), and autoimmunity against the thyrotropin receptor (TSH-R) and insulin growth factor 1 receptor (IGF-1R) [63–66].

The orbital tissue in GO is infiltrated by a variety of mononuclear inflammatory cells like CD4+ T cells, CD8+ cells, B cells, and macrophages [67]. Cell-mediated Th1 response predominates in the orbit in the early stages of GO, while the Th2 response plays a role in the later stages of the disease [68]. Interestingly, IgG4 production is controlled primarily by T helper type 2 (Th2) cells. Th2 cytokines such as interleukin-4, interleukin-10, interleukin-12, interleukin-13, and interleukin-21 enhance IgG4 production [1, 10].

The TSH receptor antibodies (TRAb) play a key role in GD and GO pathogenesis. Elevated TRAb serum concentrations are associated with the development of GO as well as the activity and severity of the disease [69–71]. The effects of the autoimmunity directed against TSH-R in orbital cells are mediated through the phosphoinositide 3-kinase/Akt, adenylyl cyclase/cAMP signaling cascades, and other signaling pathways [72]. IgG subclass depletion studies performed by Latrofa et al. indicated that TSH-R autoantibodies are predominantly IgG1 and IgG4 [73]. Many recent studies found that TRAb titers correlated with serum IgG4 concentrations in GD and GO patients [74–76].

Recent *in vitro* studies suggest that the reactivation of Epstein-Barr virus (EBV) might play a role in the development of GD and IgG4 might be one of the contributing factors in this mechanism. Nagata et al. induced EBV reactivation on peripheral blood mononuclear cells containing TRAb(+) EBV(+) cells sourced from GD patients and healthy controls. EBV reactivation resulted in TRAb production, which was significantly higher in GD patients than in controls [77]. The same group also implied that EBV reactivation induces plasma cell differentiation, rescue, and class-switched Ig production [78]. Finally, a subsequent study demonstrated that EBV reactivation on peripheral blood mononuclear cells derived from GD patients results in a higher percentage of IgG4/IgG levels in culture fluids than that from normal individuals. Moreover, *in situ* hybridization and immunohistochemistry on thyroid glands revealed that EBV-encoded small RNA1- (EBER1-) positive cells and IgG4-positive plasma cell infiltration were detected in the same areas along with lymphoid cell infiltration [79].

### 3.3. Histopathology

Studies evaluating the presence of histological features of IgG4-RD in GD patients are very scarce and deliver contradictory results. According to a study by Nishihara et al., only 11 out of 1484 thyroid glands resected from GD patients had a diffuse lymphoplasmacytic infiltration in the stroma, which is a characteristic feature of IgG4-RD and IgG4 thyroiditis. Only 5 of those 11 had IgG4-positive plasma cells in the thyroid, only one showed fibrosis, and none of the 11 specimens had obliterative phlebitis [80]. In contrast to this finding stands the study by Nagata et al., which analyzed 11 thyroid specimens of GD patients, who underwent thyroidectomy due to failure of antithyroid therapy. Seven out of the 11 analyzed cases had focal moderate infiltration of lymphocytes with lymphoid follicle formation, and 6 out of those 7 had a high number of IgG4+ plasma cells [79]. In both studies, the “high number of IgG4+ plasma cells” was defined as >10 cells in a high power field (HPF) and a ratio of IgG4(+) cells to IgG(+) cells > 40%, which is the most common criterium used in IgG4-RD [81]. However, some authors suggest using organ-specific criteria, in the case of the thyroid gland as above 20 IgG4(+) plasma cells in HPF and an IgG4(+) to IgG(+) plasma cell ratio > 30% [82]. None of the abovementioned studies found other characteristic features of IgG4-RD like storiform fibrosis or obliterative phlebitis in the analyzed thyroid gland specimens, while fibrosis was present in only one case in the cohort analyzed by Nishihara et al. [80].

While thyroid biopsies cannot be used to diagnose IgG4-RTD, the biopsy procedure is the gold standard of IgG4-ROD. It is usually obtained from the extraocular muscle, lacrimal gland, orbital mass lesion, and infraorbital nerve or a combination of these [83]. The diagnostic criteria for IgG4-related orbital disease, proposed by the Japanese Ophthalmological Society, are listed in Table 1 [84]. Many authors use slightly modified criteria with lower or higher thresholds of IgG4(+) plasma cells in HPF necessary for diagnosis, like >10 cells in the HPF threshold [81] or >100 IgG4(+) cells in HPF for the lacrimal gland as suggested by Deshpande et al. [30]. Storiform fibrosis and obliterative phlebitis, which are a rare finding in IgG4-RTD, were also reported in just around 10% and less than 10% of orbital biopsies, respectively [83].

A study by Wong et al. reported no significant IgG4 staining in 26 orbital biopsies of GO patients. This needs to be interpreted with caution as the study analyzed mostly specimens obtained from orbital fat, while only four biopsies were obtained from the lacrimal gland and none from extraorbital muscles [85]. This is confirmed by the case report of a patient with GO, in which no staining for IgG4 in the lacrimal gland and orbital fat was demonstrated but 24 IgG4(+) cells in HPF and fibrosis in an enlarged extraorbital muscle biopsy [86].

There are currently a handful of case reports describing patients with GO, and the biopsy procedure confirmed IgG4(+) plasma cell infiltration of the orbital tissue [86–88]. In those cases, biopsies were obtained most often not from the orbital fat but from the extraorbital muscle, lacrimal gland, or infiltrative orbital masses.

### 3.4. Analysis of Clinical Studies on the Role of IgG4 in Graves' Disease and Orbitopathy

There are only a few studies, often with ambiguous outcomes, assessing the role of IgG4 in Graves' Disease and even less in Graves' Orbitopathy. We summarized data from those studies in Table 2. An extensive analysis of those studies is described in the main text beneath.

Multiple studies showed higher serum IgG4 concentrations in GD patients than in healthy euthyroid controls [74–76, 89, 92].

Elevated serum IgG4 levels occur in 6.4-23% (average: 10.3%) of all patients with GD, 8.3-37.5% (average: 17.6%) of patients with GO, and 0-9.8% (average: 5.4%) of patients with GD without GO, while GO patients comprise 37.5-100% (average: 65.8%) of all GD patients with elevated IgG4 levels (Table 2). The frequency of IgG4 elevation varies depending on various factors, mostly GO prevalence, IgG4 measurement methods, and criteria used for defining abnormally high IgG4 levels. Most studies used the well-known and most widely used cut-off value adopted from IgG4-RD studies (>135 mg/dl), while some others used the 73rd [76], 75th, and 90th percentiles as cut-off values for defining abnormally high IgG4 levels [92].

According to the first and largest (109 patients) study on this subject published in 2014 by Takeshima et al., patients with GD and elevated IgG4 were older than those with normal IgG4 levels (54.7 ± 6.2 vs. 43.4 ± 15.4 years), respectively. [74] However, subsequent smaller studies failed to show a statistically significant difference in age. Interestingly, another study found that patients with GD and high IgG4 levels had lower mean age at GO onset (26.33 ± 2.58 vs. 45.83 ± 13.70 years, *p* < 0.001) [92].

In a study by Yu et al., female sex, smoking history, and levels of IgG, IgG4, triiodothyronine (T3), free thyroxine (FT4), TRAb, and antithyroglobulin antibodies (TgAb) were associated with GO development in univariate analysis. However, the serum IgG4 concentration was found to be the only independent factor associated with GO development in multivariate analysis (*p* = 0.046, odds ratio (OR) = 1.351, and 95% CI: 1.006–1.815). The same study found that IgG4 levels and the IgG4/IgG ratio are elevated in the moderate-to-severe orbitopathy group compared with the group with mild orbitopathy and in the active GO group compared with the inactive GO group. IgG4 levels were also associated with the Clinical Activity Score (CAS), which is an often employed and renowned classification used to grade the severity of GO. Average IgG4 levels were 46.5 ± 15.9 vs. 74.1 ± 9.4 vs. 93.9 ± 19.9 mg/dl (*p* = 0.001) for groups of patients with a CAS of 1, 2, and 3, respectively. IgG4/IgG ratios were 3.6 ± 1.6 vs. 5.3 ± 2.0 vs. 6.0 ± 1.5% (*p* = 0.033) for groups with a CAS of 1, 2, and 3, respectively [76].

In another study, IgG4 levels were observed to increase in concordance with CAS and IgG4 levels and were also higher in the GO group compared with GD patients without GO (101.5, range: 15-366 vs. 51, range: 13-203 mg/dl; *p* = 0.024) [89]. Conversely, the studies by Takeshima et al. and Sy and Silkiss failed to demonstrate any statistically significant relationship between IgG4 levels and CAS or the prevalence of GO [74, 90]. This might be, however, due to the small sample size of GO patients with elevated IgG4, thus a lower statistical power.

GD affects mostly women, but thyroid eye disease in general tends to be more severe in older patients and men. In a study by Perros et al., the female to male ratio was 9.3 in patients with mild orbitopathy, 3.2 in those with moderate orbitopathy, and only 1.4 in those with severe orbitopathy. This was often related to the fact that men tend to be heavy smokers [94, 95], and cigarette smoking is a well-established risk factor for GO development [96]. However, another factor to take into consideration is that IgG4-RD shows a male predominance with an overall male : female ratio of 8 : 3 [97]. A male predominance in GD and GO patients with high serum IgG4 concentration was found by Torimoto et al. [91], while Martin et al. found higher serum IgG4 concentrations in men *de novo* diagnosed with GD (225.69 ± 93.19 vs. 140.82 ± 93.97 mg/dl) [92]. However, other studies did not show statistically significant differences in gender; thus, further research on larger populations is required to determine the significance of those findings.

Another thing indirectly linking GD to IgG4-RD is the peripheral blood eosinophilia. Approximately 40% of patients with IgG4-RD have eosinophilia [98, 99], which is not related to atopy [100]. No such finding was reported in GD; however, patients with GD and elevated IgG4 levels were found to have higher peripheral blood eosinophil counts than those with normal IgG4 levels -363 ± 354/mm^2^ and 136 ± 122/mm^2^, respectively [91].

In one study, patients with immunoglobulin G4 levels above the 75th percentile were reported to have higher concentrations of antithyroid peroxidase antibodies (TPOAb) (*p* = 0.01) and TgAb levels (*p* = 0.006) [92]. TPOAb correlated with IgG4 and IgG levels in another study [75]. However, those findings were not confirmed by other authors.

Regarding GD treatment with antithyroid drugs, patients with IgG4 levels above the 75th percentile require a shorter duration of the first methimazole treatment cycle than patients with immunoglobulin G4 below the 75th percentile. At diagnosis, patients with immunoglobulin G4 levels above the 90th percentile tend to have lower total T3 (252.00 ± 55.94 vs. 361.68 ± 160.65 ng/dl, *p* = 0.001) and significantly higher FT4/FT3 ratios (0.95 ± 0.86 vs. 0.46 ± 0.27, *p* = 0.027) than patients with IgG below the 90th percentile [92]. Patients with elevated serum IgG4 concentrations more often become hypothyroid after treatment with antithyroid drugs [74]. Some studies have shown that GD patients with elevated IgG4 levels tend to have lower echogenicity of the thyroid on ultrasound examination [74, 91].

Data on time-dependent and treatment-dependent changes of IgG4 levels in GD and GO patients are incredibly scarce. In spite of some case reports, to date, there is only one longitudinal study, which followed 9 patients with GD (two with elevated IgG4 and 7 with normal values at the start of the study) for a mean of 10.4 ± 8.0 months. It described a return of IgG4 levels elevated above 135 mg/dl to normal values after antithyroid treatment; however, one of the patients with normal baseline IgG4 levels had an increased level after antithyroid treatment [93].

According to a study by Luo et al., patients with GO with an elevation of IgG4 present most commonly with bilateral muscle thickening (in 7/8 cases), which affects the medial rectus muscle, the inferior rectus muscle, and relatively often the lateral rectus muscle (in 7/8, 6/8, and 5/8 cases, respectively). Infraorbital nerve thickening and lacrimal gland involvement, which are key features of IgG4-ROD, were less prevalent (1/8 and 2/8 cases, respectively) [75]. All those findings, despite the substantial lateral muscle involvement, are similar to the findings described in the general population of GO patients, which are enlargement of the inferior rectus and medial rectus muscles with muscle tendon sparing [101, 102]. On the other side, elevated serum IgG4 levels were associated with bilateral disease in IgG4-ROD and the lateral rectus muscle is the most commonly extraorbital muscle affected in IgG4-ROD [103].

The diagnostic dilemma of distinguishing between GO and IgG4-ROD was described in a very detailed and informative case series by Tooley et al. [88]. In this study, patients were divided into three categories: “Possible past GO without expected clinical course,” “IgG4-ROD initially misdiagnosed as GO,” and “Possible concomitant GO and IgG4-ROD.” There is, however, a strong possibility that at least some of those patients could be reclassified as “Graves' Orbitopathy with elevated IgG4 levels,” especially those with a confirmed GO diagnosis, elevated serum IgG4 levels, bilateral disease, enlargement of multiple eye muscles, especially the medial rectus muscle, and no other systemic IgG4-RD manifestations.

To date, no trials assessing response to standard or potentially different treatment regimens in GO with elevated IgG4 have been conducted. However, based on studies on IgG4-RD, IgG4-ROD, GO in general, and some case reports, treatment with glucocorticoids is the first line of therapy. However, the disease has a relatively high risk of recurrence when the dose is tapered, and in these cases, other immunosuppressive regimens might be helpful. Currently, also, rituximab is considered a primary therapeutic strategy, which allows steroid discontinuation and relieves signs and symptoms [88, 104–107]. Rituximab acts by depleting CD20^+^ B cells. CD20^+^ B cells are progenitors to plasmablasts. Plasma cells that produce IgG4 are usually short-lived, and they undergo naturally programmed cell death within a couple of weeks. After this process is completed, the plasma cell pool cannot be repeated because the B cells, which are their precursors, are already depleted. This explains also why it usually takes rituximab a couple of weeks to work [104, 108]. Fibrotic changes decrease the response to the therapy. This underscores the importance of early diagnosis and fast introduction of proper therapy.

## 4. Other Inflammatory Eye Diseases to Be Included in the Differential Diagnosis

Symptoms of IgG4-ROD may overlap those seen in other diseases, though in most patients, both eyes are affected. Likewise, CT or MRI scans of the orbital region also present unspecific features [109]. The presence of the high amount of IgG4+ cells in the biopsy cannot be the only feature in setting diagnosis, as they are also detected in other diseases like granulomatosis with polyangiitis (GPA), eosinophilic granulomatosis with polyangiitis (EGPA, Churg-Strauss syndrome), xanthogranulomatous disease, or lymphoma [110–112].

In the study by Wong et al., they collected orbital biopsies from patients diagnosed with nonspecific orbital inflammation (NSOI; *n* = 42), GPA (*n* = 6), sarcoidosis (*n* = 12), and thyroid eye disease (*n* = 26). With the IgG4 plasma cell threshold set at >10/HPF, positive staining was present in patients with GPA (83%), NSOI (38%), and sarcoidosis (42%). Only a few cases had more than 30 IgG4-PC in HPF. In contrast to IgG4-RD, in examined biopsies, storiform fibrosis or obliterative phlebitis was not detected [85].

Lymphoma is the most common malignancy of the ocular adnexa [112]. Cheuk et al. reported a case series of 6 patients who probably developed ocular adnexal lymphoma from IgG-related chronic sclerosing dacryoadenitis (three patients had a previous histopathological diagnosis of IgG4-RD, and three did not). Biopsies from all cases showed infiltration of IgG4 plasma cells. However, the authors discussed that it is unclear if lymphoma arises from preexisting IgG4-ROD or it is *de novo* IgG4 MALT lymphoma [113].

Peng et al. reported a similar case of lymphoma in a 44-year-old patient who complained of bilateral proptosis for several years. Before admission to the hospital, he experienced rapid loss of vision in the right eye. During hospitalization, the patient underwent right eye exenteration and tumor resection for the left eye. Based on the chronic history, serum IgG4 concentrations, IgG4 plasma cell infiltration, and acute deterioration, the authors supposed transformation of IgG4-ROD to diffuse large B cell lymphoma [112].

In summary, it should be noted that IgG4+ plasma cells can be found in the histopathological examination of various diseases. Even though the histopathological examination is the gold standard in the diagnostic process of IgG4-ROD, its results should always be interpreted together with the patient's clinical symptoms, comorbidities, and outcomes of other tests in order to avoid misdiagnosis.

## 5. Conclusions

In this review, we described the pathophysiological, histopathological, and clinical features of Graves' Disease and Graves' Orbitopathy with elevated IgG4 levels, which show many distinct characteristics compared to the classic forms of Graves' Disease and Orbitopathy.

Based on current research, characteristic features of Graves' Disease with elevated IgG4 levels are lower echogenicity of the thyroid gland on ultrasound examination, peripheral blood eosinophilia, higher prevalence of orbitopathy, and better response to antithyroid drugs with a tendency to develop hypothyroidism.

Characteristic features of Graves' Orbitopathy with elevated IgG4 levels include younger age at orbitopathy diagnosis, and more severe course of the disease with higher CAS. Such patients should also have a good response to rituximab therapy, but this has not yet been studied in clinical trials.

It is prudent to remember that GO patients with elevated IgG4 can also present with an overlap of classical GO and IgG4-ROD features. Systemic manifestations of IgG4-RD are, however, not typical.

At the moment, there is not enough evidence to call this entity “IgG4-related Graves' Disease”; thus, the term “Graves' Disease/Graves' Orbitopathy with elevated IgG4 levels” seems more appropriate.

Future research should focus on developing longitudinal prospective studies, especially assessing responses to different treatment regimens. More studies assessing histopathological features of orbital biopsies (especially extraorbital muscle biopsies) and resected thyroid samples are also needed.

We strongly recommend considering the diagnosis of Graves' Orbitopathy with elevated IgG4 in patients with an established diagnosis of Graves' Disease, elevated serum IgG4 levels, and clinical features of ophthalmic disease overlapping with those of IgG4-related orbital disease.

## Figures and Tables

**Table 1:** Diagnostic criteria for IgG4-related ophthalmic disease, based on the criteria established in 2014 by the Japanese Ophthalmological Society [84].

(A) Imaging studies: enlargement of the lacrimal gland, trigeminal nerve, or extraocular muscle; masses, enlargement, or hypertrophic lesions in various ophthalmic tissues (B) Histopathologic examination: marked lymphocytic and plasmacytic infiltration, sometimes fibrosis. Often the presence of a germinal center. IgG4+ plasmacytes are found and meet the following criteria: a ratio of IgG4+ cells to IgG+ cells of 40% or above or more than 50 IgG4+ cells per HPF (×400) (C) Blood test: elevated serum IgG4 concentration (>135 mg/dl)

Based on the fulfillment of the criteria mentioned above, the probability of the diagnosis is defined as follows: “Definitive diagnosis”—when criteria A, B, and C are fulfilled “Probable diagnosis”—when A and B are fulfilled “Possible diagnosis”—when A and C are fulfilled

**Table 2:** Summary of clinical studies on the role of IgG4 in Graves' Disease and Graves' Orbitopathy.

Study author, year of publication	Prevalence of elevated IgG4 in GD patients overall	Prevalence of elevated IgG4 in GD patients without GO	Prevalence of elevated IgG4 in GO patients	Prevalence of GO in the GD group with elevated IgG4	Age in the nonelevated IgG4 group (mean ± SD)	Age in the elevated IgG4 group (mean ± SD)	Sex distribution in the nonelevated IgG4 group (M/F (*M*%))	Sex distribution in the elevated IgG4 group (M/F (*M*%))	Average IgG4 levels in the nonelevated IgG4 group (mg/dl)	Average IgG4 levels in the elevated IgG4 group (mg/dl)	Criteria used to define elevated IgG4	IgG4/IgG ratio in the nonelevated IgG4 group (%)	IgG4/IgG ratio in the elevated IgG4 group (% ± SD)
Takeshima et al. 2014 [74]	7/109 (6.4%)	4/80 (5%)	3/29 (10.3%)	3/7 (42.3%)	43 ± 15.4	54.7 ± 6.2	14/88 (15.9%)	1/6 (14.2%)	39.6 ± 27.6	175 ± 44.5	>135 mg/d	3.2 ± 2.2	12.7 ± 4.5
Bozkirli et al. 2015 [89]	15/65 (23%)	3/33 (9.1%)	12/32 (37.5%)	12/15 (80%)	n.a.	n.a.	n.a.	n.a.	n.a.	n.a.	>135 mg/d	n.a.	n.a.
Sy and Silkiss^∗^ 2016 [90]	n.a.	n.a.	2/24 (8.3%)	n.a.	50.79 ± 14.75	43.5 ± 16.26	5/22 (22.7%)	0/2 (0%)	51.95 ± 33.19	160 ± 9.9	>135 mg/dl	5.02 ± 3.34	8.15 ± 431
Torimoto et al. 2017 [91]	5/72 (6.9%)	n.a.	n.a.	n.a.	41.9 ± 15.6	43 ± 18.7	16/67 (23.9%)	4/1 (80%)	37.5 ± 27.1	206 ± 115.8	>135 mg/dl	2.8 ± 1.9	10.6 ± 3.3
Martin et al. 2017 [92]	8/80 (10%)	5/51 (9.8%)	3/29 (10.3%)	3/8 (37.5%)	44.85 ± 15.66	39 ± 15.09	12/72 (16.7%)	3/8 (37.5%)	139.56 ± 88.71	311.36 ± 21.37	>90th percentile	n.a.	n.a.
Yu et al. 2017 [76]	6/64 (9.4%)	0/58 (0%)	6/22 (27.7%)	6/6 (100%)	37.2 ± 4.6	32.3 ± 9.5	16/62 (25.8)	2/6 (33.3%)	54.9 ± 17.5	98.9 ± 9.8	>86.4 mg/dl	4.2 ± 1.9	6.1 ± 1.13
Hiratsuka et al. 2020 [93]	2/28 (7.1%)	n.a.	n.a.	1/2 (50%)	45.5 ± 6.0	66 and 70	5/23 (17.8%)	0/2 (0%)	n.a.	163.5 and 214.4	>135 mg/dl	n.a.	15 and 14.8
Luo et al.^∗^ 2020 [75]	n.a.	n.a.	8/57 (14%)	n.a.	46.69 ± 10.87	50.62 ± 12.96	19/39 (38.78%)	1/6 (16.67%)	63.45 ± 37.02	187.62 ± 43.05	>135 mg/dl	n.a.	13.55 ± 2.74
Summary	43/418 (10.3%)	12/222 (5.4%)	34/193 (17.6%)	25/38 (65.7%)	—	—	87/373 (18.9%)	11/31 (26.2%)	—	—	—	—	—

^∗^Studies that assessed only the role of IgG4 in GO. n.a.: data not available; SD: standard deviation.

## Data Availability

All data were obtained from published articles, which are cited in the bibliography.
